# Measuring motivation among close-to-community health workers: developing the CTC Provider Motivational Indicator Scale across six countries

**DOI:** 10.1186/s12960-020-00495-7

**Published:** 2020-08-01

**Authors:** Frédérique Vallières, Maryse Kok, Ilias Mahmud, Malabika Sarker, Philippa Jeacocke, Robinson Karuga, Licia Limato, Aschenaki Z. Kea, Kingsley Chikaphupha, Mohsin Sidat, Brynne Gilmore, Miriam Taegtmeyer

**Affiliations:** 1 Trinity Centre for Global Health, School of Psychology, Trinity College Dublin, 7-9 Leinster Street South, Dublin 2, Ireland; 2 KIT Royal Tropical Institute, Mauritskade 64, 1092 AD Amsterdam, The Netherlands; 3 James P Grant School of Public Health, BRAC University, 68 Shahid Tajuddin Ahmed Sharani, Mohakhali, Dhaka, 1212 Bangladesh; 4 Liverpool School of Tropical Medicine, Pembroke Place, Liverpool, L3 5QA UK; 5 LVCT Health, Argwings Kodhek Rd, Nairobi, Kenya; 6 Eijkman Institute for Molecular Biology, Jl. Diponegoro No.69, Jakarta, Indonesia; 7 Reach Ethiopia, Box 303, Hawassa, Ethiopia; 8 Research for Equity and Community Health Trust, Box 1597, Lilongwe, Malawi; 9 Department of Community Health, University Eduardo Mondlane, Maputo, Mozambique; 10 UCD Centre for Interdisciplinary Research, Education and Innovation in Health Systems, School of Nursing, Midwifery and Health Systems, University College Dublin, Dublin, Ireland

**Keywords:** Close-to-community health workers, Community health workers, Motivation, LMICs

## Abstract

**Background:**

Close-to-community (CTC) health service providers are a cost-effective and important resource in the promotion of and increasing access to health services. However, many CTC provider programmes suffer from high rates of de-motivation and attrition due to inadequate support systems. Recent literature has identified the lack of rigorous approaches towards measuring and monitoring motivation among CTC providers as an important gap. Building on scales used in previous studies, we set out to develop a short, simple-to-administer scale to monitor and measure indicators of CTC provider motivation across CTC programmes implemented in six countries: Ethiopia, Kenya, Malawi, Mozambique, Indonesia, and Bangladesh.

**Methods:**

We used focus group discussions (*n* = 18) and interviews (*n* = 106) conducted with CTC providers across all six countries, applying thematic analysis techniques to identify key determinants of motivation across these contexts. These themes were then used to carry out a systematic search of the literature, to identify existing scales or questionnaires developed for the measurement of these themes. A composite 24-item scale was then administered to CTC providers (*n* = 695) across the six countries. Survey responses were subsequently randomly assigned to one of two datasets: the first for scale refinement, using exploratory techniques, and the second for factorial validation. Confirmatory factor analysis was applied to both datasets.

**Results:**

Results suggest a 12-item, four-factor structure, measuring community commitment, organisational commitment, job satisfaction, and work conscientiousness as common indicators of motivation among CTC providers across the six countries.

**Conclusions:**

Consistent with previous studies, findings support the inclusion of job satisfaction, organisational commitment, and work conscientiousness within the CTC Provider Motivation Indicator Scale. In addition, findings further supported the addition of a fourth, community commitment, sub-scale. Practical applications of the revised scale, including how it can be applied to monitor motivation levels within CTC provider programming, are discussed.

## Background

Close-to-community (CTC) health service providers offer a crucial link between communities and health systems, catering to individuals that otherwise remain beyond the reach of formal health facilities [1]. Compared to formal health workers, CTC providers tend to lack professional training, are embedded in the community, and are often volunteers [2]. Results from systematic reviews suggest that CTC providers are not only cost-effective [3], but also positively influence the uptake of immunisations [4], increase appropriate maternal and newborn care practices [5], and prevent the spread of communicable diseases [6, 7]. Consequently, CTC providers are widely used to address the severe shortage of human resources for health in low- and middle-income countries (LMICs) [8] and are often cited as a key strategy towards achieving universal health coverage.

Despite CTC providers being largely effective in delivering health services to communities, they remain particularly vulnerable to high rates of de-motivation and attrition [9, 10]. Frequent CTC provider turnover places considerable strain on health systems, due to the cost and time necessary to recruit, train, and supervise new CTC providers [11]. To remain motivated, performing and retained in the long-term, CTC providers require effective management, training, supervision, and both financial and non-financial incentives [12, 13]. In this way, CTC providers are not immune to the same underlying factors that negatively impact the motivation of more highly qualified, salaried, formal health workers. The World Health Organization’s (WHO) most recent *Global Strategy on Human Resources for Health: Workforce 2030* emphasises the importance of implementing policies that promote decent working conditions for health workers as key determinants of health worker retention and motivation [14]. Therefore, and as Banek et al. [15] rightly point out, if increasingly complex community interventions are going to be part of the solution to inadequate access to primary care, greater attention as what *motivates* individuals within health systems is required.

Prominent in the fields of psychology and behavioural economics, the study of motivation is largely concerned with the drives or forces acting on or within an individual that result in the initiation or continuation of a behaviour [16]. Here, motivation is therefore considered in terms of the factors that are known to influence whether or not health workers continue to deliver healthcare services, as the displayed behaviour of interest. Seen as a key determinant of desired human resource management outcomes, evidence suggests that the motivation of CTC providers is associated with both individual and programme performance [17, 18]. Specifically, motivation is thought to both directly impact on performance and mediate or moderate the effect of interventions targeting CTC provider performance [19]. For example, motivation is seen as an important factor in mitigating attrition rates [20, 21], preventing high health worker turnover [22], and promoting better quality of care [23]. Similarly, loss of motivation among health workers is associated with higher rates of burnout [24], poor or unsupportive supervision [25, 26], lack of remuneration [27], and insufficient resources necessary for CTC providers to complete their tasks [28]. Recognising the important role motivation plays in the retention and performance of CTC providers, recent literature has called for more systematic, rigorous approaches to the development of scales to measure motivation [29]. Current challenges with measuring and conceptualising motivation have contributed to the inconsistent and therefore incomparable measurement of motivation within the CTC provider literature. Moreover, there is an ongoing debate as to whether CTC provider programmes should seek to measure motivation itself, as a direct measure, or whether to assess CTC provider motivation using indirect, or proxy, measures of other indicators closely related to motivation [29].

As a psychological construct, motivation is not directly observable, and issues arise when one attempts to measure it [19, 30]. The field of psychometrics, concerned with the development of instruments that approximate theoretical or *latent constructs* (i.e. motivation) by means of measuring observable variables (i.e. responses on a questionnaire), therefore serves as an important resource to help address these gaps. Recent years have seen a greater application of psychometrical methods towards the development of scales to measure human resource management factors among health workers. Factor analysis techniques have been applied to the development of job satisfaction [31, 32], work environment [33, 34], and perceived supervision [35] scales for health providers. Tools have also been developed as either direct [36] or proxy measures of health worker motivation [2, 19, 37–41]. While these studies have undoubtedly contributed to the availability of tools available to CTC provider programmes, many of these studies [2, 19, 37, 39–41] use exploratory factor analysis (i.e. principal component analysis) techniques as an important first step in scale development. A limitation of exploratory factor analysis, however, is that it is a data-driven process. As a result, little is known about the validity of the factor structures of these tools outside of the contexts where they were first derived. Confirmatory factor analysis, on the other hand, allows one to test and subsequently falsify or confirm the factor structure of a scale previously developed using more exploratory techniques [42].

The aim of this study was therefore to build on the work of existing motivation measures to develop, and subsequently validate using confirmatory factor analysis, an easily translatable, short scale that could quickly and easily be administered and scored to regularly monitor and measure indicators of motivation of CTC providers working across the six countries of a research programme. This 5-year healthcare research programme aimed at supporting and strengthening the vital work of CTC providers in two countries in Asia (Bangladesh and Indonesia) and four countries in sub-Saharan Africa (Kenya, Mozambique, Malawi, and Ethiopia).

## Methods

### Procedures and participants

Borghi et al. put forward a series of steps on how to measure health worker motivation in LMICs. First, they suggest engaging in a conceptualisation phase, whereby observable items of the latent dimension of motivation are identified. This first step is aligned to previous scale development studies [38, 43], which used focus group discussions and/or key informant interviews to identify potential dimensions, or factors, of the latent construct of interest within that context. They then suggest a tool development and testing tool phase, whereby identified items are subject to reliability and validity testing. Therefore, and aligned to these and other best-practice recommendations, the current study sought to (i) identify the factors that are associated with the motivation of CTC providers in the LMICs participating in the programme, (ii) identify and adapt existing scales or tools designed to measure these factors associated with motivation among health workers in LMICs, and (iii) develop and assess the factorial validity of a new scale to assess motivational indicators among a heterogeneous sample of CTC providers working in LMICs. These three objectives were achieved using a cross-sectional, mixed-methods approach, over three distinct phases of the research.

During phase I, we reviewed the transcripts of focus group discussions (FGDs) (*n* = 18) and semi-structured interviews (*n* = 106) conducted with CTC providers to elucidate common factors that contribute to the motivation of CTC providers across all six contexts. CTC providers interviewed included both males and females in each country, with the exception of Ethiopia and Indonesia, where CTC providers are all female. All CTC providers were aged over 18 and were conveniently sampled, based on their involvement in CTC provider programmes. A full overview of interviews and FGDs, conducted in each country, by informant type, is provided in Additional file 1 and is also published elsewhere [44].

Common factors identified through the FGD and semi-structured interviews formed the basis of the search strategy for phase II, which consisted of a search of the literature to identify a list of potential items used in previous studies for the measurement of these emergent factors. In addition, phase II further served to identify other motivation-related factors (and their related items) that did not emerge from phase I, but which are commonly measured within the literature on CTC motivation. Together, phases I and II therefore led to the development of an initial pool of test items to be taken forward in phase III: the tool refinement and construct validation phase.

The initial pool of items was administered to 695 of the 710 CTC providers taking part in the programme. Fifteen CTC providers were unavailable to participate at the time of the survey, as they were either on leave or away for a training or scheduled workshop. To ensure a widespread representation of CTC providers, CTC providers were recruited in consultation with either national ministries of health (Bangladesh, Malawi, Mozambique, Kenya), regional (Ethiopia), or district-level health management teams (Indonesia) and based on the presence of a functioning CTC programme in these districts. A summary of CTC provider characteristics who took part in phase III by country is presented in Table 1. Consistent with our objective to develop a scale that could be used across a wide range of CTC providers, the CTC providers across the six countries varied substantially in terms of gender, education level, remuneration, and how well the respective cadres are integrated into the formal health system.

**Table 1:** Summary of CTC provider demographics and sampling methods employed across all six study locations

	Bangladesh	Ethiopia	Indonesia	Kenya	Malawi	Mozambique
Close-to-community provider acronym and basic typology	CTC health provider (volunteers)	HEW (government payroll)	Village midwives (government payroll)/kaders (volunteers)	CHEW (government payroll)/CHV (volunteers)	HSA (government payroll)	APE (volunteers)
Sampling method	Convenience	Convenience	Convenience	Convenience	Random	Convenience
Language	Bangla	Amharic	Basha-Indonesia	English, Swahili, Kamba	Chichewa	Portuguese, Ronga, Changane
Sample size	119	108	230	51	124	78
Sex (female, %)	75.6	100	97.0	76.5	38.7	61.5
Completed secondary education (%)	12.3	99.1	28.3	34.1	79.5	0

*CHW* community health worker, *CTC* close-to-community, *HEW* health extension worker, *CHEW* community health extension worker, *HSA* health surveillance assistant, *APE* Agentes polivalentes elementares, *SD* standard deviation

The initial version of the tool was translated into seven languages (Bangla, Kiswahili, Kamba, Bahasa-Indonesia, Chichewa, Portuguese, and Amharic), back-translated, and compared to the original English version. It was then piloted with five to 10 CTC providers in each context, and small adjustments to the language and terminology were made before the tool was administered by trained enumerators in health facilities/community outposts. Items were scored using a 5-point Likert scale, anchored by strongly disagree (= 1) and strongly agree (= 5). Phases I and II took place between July and September 2013, and phase III took place between October 2014 and May 2015.

### Specific study procedures and data analysis

#### Phase I—Interviews and focus group discussions with CTC providers

Inter-country discussions were conducted among researchers who had conducted the FGDs and interviews as part of phase I. The researchers were selected on the basis that they had interacted formally with additional key stakeholders (supervisors, clients of CTC providers) and with implementers of community health programmes in the six countries. The analysis was conducted inductively, according to major emerging themes and sub-themes. The initial number of factors influencing CTC provider motivation generated in phase I was used to inform the search strategy carried forward in phase II.

#### Phase II—Literature review

A review of available published and grey literature on motivational theory, motivational determinants, motivational indicators, and existing motivational tools, with a focus on CTC providers, was conducted. Three databases were searched including SCOPUS, Medline, and CINAHL, and relevant papers were identified. Citation searches as well as searches of the reference lists of relevant papers, conference abstracts, and ongoing consultations with experts were then performed to identify further papers of interest. Papers were included if they proposed scales or items to measure motivational indicators, regardless of whether these had been formally validated. These scales were subsequently mapped onto the results of phase I to identify (i) common items that had previously been used to measure the motivation-related factors identified in phase I and (ii) any additional motivation-related factors (and their associated items) that did not emerge in phase I but that have been previously used to measure motivation. The items identified in phase II were then synthesised with the results of phase I to derive an initial pool of items to take forward for refinement and validation during phase III.

#### Phase III—Tool refinement and validation

Refinement and validation of the initial scale were conducted using confirmatory factor analysis (CFA) methods. In line with best practice [45], the 695 CTC provider cases were randomly assigned to one of two separate data files using SPSS (version 24.0). The first file (*n* = 345) was used to apply exploratory techniques within measurement modelling procedures [46]. In this phase, the hypothesised latent structure of the initial scale was explored, and items meeting the following criteria were considered for exclusion: items with coefficients of < 0.3, items with non-significant parameters (*P* ≤ 0.05), and cross-loading items (i.e. items that loaded considerably onto more than their designated factor) [47]. Item reduction also occurred by consulting modification indices, which provide suggestions of additional items that could be removed (i.e. items with covarying residuals) or which parameters can be freed in order to reduce the chi-square and thus improve model fit [48].

The reduced set of items was then carried forward and a second confirmatory factor analysis was conducted with the second randomly generated data file (*n* = 350). All factor analyses were conducted using MPlus [49] version 6.0 with robust maximum likelihood (MLR) estimation. Model fit was determined using standard recommendations [50] whereby acceptable model fit was indicated by a chi-square-to-degree of freedom ratio (*χ*^2^:df) less than 3:1, Comparative Fit Index (CFI) and Tucker-Lewis Index (TLI) values > .90, a root mean square error of approximation (RMSEA) value < .08, and a standard root mean square residual (SRMR) value < .08. Internal reliability was assessed using a composite reliability analysis [51].

## Results

### Phase I—Interviews and focus group discussion with CTC providers

Results from the interviews suggested a number of common, inter-related factors influencing the motivation of CTC providers across the six countries. These were categorised under five motivational factors: organisational commitment, extrinsic job satisfaction, community commitment, intrinsic job satisfaction, and work conscientiousness. Though there were variations in what was understood by ‘organisation’, most CTC providers saw commitment to the organisation as associated with motivation. Overall, CTC providers felt proud to work for their organisation. However, when asked what makes them feel demotivated in their work, participants frequently noted a lack of recognition and support offered by their respective ministries of health:

…we are the eyes of the Ministry of Health in this country and we also are a shield when it comes to protecting the community’s health. [The community] people always come to us before they go anywhere and yet the authorities seem not to care about us at all… Health Surveillance Assistant, FGD, Malawi

The Health Extension Programme is hard working and very exhaustive, but the government does not realize it. Health Extension Worker, Interview, Ethiopia

Job satisfaction was also identified as a key determinant of motivation, whereby extrinsic job satisfaction factors including a lack of resources, poor remuneration, lack of appreciation for CTC providers’ workload and role, irregular or fault-finding approaches to supervision, insufficient supplies and logistical support, and limited career perspectives were all identified as particularly demotivating.

Now the Kaders don’t have motivation to do the vaccination because there is no support for their transportation. Kader CHW, Interview, Indonesia

It is not fair, it is not fair, it is not fair [the supervision], because it does not motivate me… it mostly entails administration because they just come for fault finding. Community Health Extension Worker, Interview, Kenya

On the other hand, extrinsic job satisfaction factors such as recognition and gaining knowledge through training and supportive supervision were identified as having a positive impact on motivation.

In fact in 2005 EC we made our kebele (community) free of open field defecation. During that time we receive recognition from our kebele and from woreda health office. This motivates me to work hard further. Community Health Worker, Interview, Ethiopia

Also with the supervision we can communicate directly about our needs. The supervision increases my motivation and also improve my knowledge. It keeps me motivated. Kader CHW, Interview, Indonesia

Community commitment emerged as another key theme related to the motivation of CTC providers. CTC providers across the six countries felt part of the communities they worked in and had a sense of belonging to these communities. They often regarded community members as their ‘families, sisters, or brothers’ who inspire them in their work. Support and recognition from the side of the community were reported to increase motivation.

…We feel well appreciated by the people we help and this encourages us to continue doing our work. This appreciation makes us feel proud that we are doing a good job. Health Surveillance Assistant, FGD, Malawi

Delivery in hospitals is very expensive. My motivation behind working here [in the community] is to serve the people [in the community]. CTC Provider, Bangladesh

If they (community) listen to me then I’m motivated to do my job. An award like trophy can be bought but their gratitude as the appreciation cannot be bought...... I have sacrificed my time with my family to work for the community. And if they still do not want to listen to me that makes me sad. If they listen it makes me happy, because it means I’m successful and vice versa. Community Health Worker, Interview, Indonesia

Community commitment was also related to intrinsic job satisfaction and often resulted from seeing a positive impact in the community as a result of the work of the CTC provider.

It gives me pleasure when I see malnourished children getting well after receiving treatment from me. Health Extension Worker, Interview, Ethiopia

Work conscientiousness was the last theme, or motivational indicator, identified. Both personally and culturally driven, it was described in different ways across individuals and contexts. The factors that influenced work conscientiousness included a desire to help those in need, a desire to improve community health, and worshiping God through the work as a CTC provider. In all cases, it was related to a sense of responsibility and taking pride in one’s work and was positively associated with motivation.

Since my childhood I was interested to serve the humanity, for this reason I choose this profession. At the same time I noticed the high demand of Ayurvedic and Unani [herbal] medicine along with these [52] medicines, so for this reason I joined this profession. Informal CTC provider – drug store salesman, Interview, Bangladesh

I’m proud with my job as a kader, because I realize that this job is a social work. When they were asking me to become a kader, I thought that I would get an experience and give my worship to God as well. Kader, Interview, Indonesia

### Phase II—Literature review

A total of four tools were identified in the literature review: one focused on the motivation of community health workers volunteering across three family planning programmes in Uganda [2]; one focused on mid-level cadres and auxiliary staff at primary health care level in rural Burkina Faso, Ghana, and Tanzania [39]; and two focused on facility-based public-sector health workers in Kenya [37] and Georgia and Jordan [40], respectively. Consistent with the factors emerging from phase I, these tools also measured organisational commitment, job satisfaction, and work conscientiousness. In addition, the literature review suggested the addition of two additional factors: burnout and general motivation.

However, and whereas phase I highlighted the prominence of community commitment in CTC provider’s motivation, this construct was found to be absent in the existing tools identified from the literature review. Table 2 summarises the source for each of the factors included in the initial questionnaire and the initial set of 24 items identified to measure each of these factors. Items were identified based on common items used across previously existing scales and the results of phase I (i.e. in the case of community commitment). The first two phases of the research therefore led to the development of an initial pool of 24 items measuring seven motivational indicators: the five motivational factors identified in phase I and two additional factors identified in phase II.

**Table 2:** Factors, items, and uses of the initial 24-item CTC Provider Motivational Indicator Scale

Factor	Source(s)/use(s)	Items	Comments
Organisational commitment	(Mbindyo et al., 2009, Prytherch et al., 2012, Mutale et al., 2013, Penn-Kekana et al., 2005, Bennett et al., 2001)	I am proud to be working for my organisation as a CTC provider (OC1)	In each context, the word ‘organisation’ was adapted from ‘hospital’ or ‘health facility’ used in other questionnaires. In most of the six countries, the organisation was identified as the Ministry of Health.
	(Mutale et al., 2013, Mbindyo et al., 2009, Penn-Kekana et al., 2005, Bennett et al., 2001, Brunie et al., 2014)	I feel very little commitment to my organisation (OC2)	
	(Mutale et al., 2013, Mbindyo et al., 2009, Bennett et al., 2001)	My organisation really inspires me to do the very best on the job (OC3)	
Extrinsic job satisfaction	Phase I findings as well as adapted from Mutale et al., 2013, Mbindyo et al., 2009	I am satisfied with the support I receive from my colleagues (JS1)	Satisfaction with the support from colleagues, community, and supervisor all identified as important during phase I data analysis.
	(Bennett et al., 2001, Prytherch et al., 2012)	I am satisfied with the opportunities for further training I have on this job (JS2)	
	Phase I findings	I am satisfied with the community thanks and recognition I receive for my work (JS3)	
	Phase I findings and adapted from Prytherch et al., 2012, Brunie et al., 2014, Penn-Kekana et al., 2005, Bennett et al., 2001	I am satisfied with the financial support I receive from doing my work (JS4)	
	Phase I findings and adapted from Mbindyo et al., 2009, Prytherch et al., 2012, Mutale et al., 2013, Penn-Kekana et al., 2005, Brunie et al., 2014, Bennett et al., 2001	I am satisfied with the support I receive from my supervisor (JS5)	
Community commitment	Phase I findings	I am proud to be working for my community (CC1)	None of the motivation scales identified during phase II incorporated a community commitment factor. The inclusion of this factor is therefore based on the findings from phase I only, borrowing from the language used in items measuring organisational commitment.
		My community inspires me to do the very best I can for them (CC2)	Brunie et al. (2014) do allude to the community as an important factor, but all item statements are combined with a remuneration component (i.e. ‘I enjoy working in my community, even if it is without pay’; ‘I do not think it makes good sense for me to spend any time working in my community without payment’).
Intrinsic job satisfaction	(Mbindyo et al., 2009, Mutale et al., 2013, Penn-Kekana et al., 2005, Brunie et al., 2014, Prytherch et al., 2012, Bennett et al., 2001)	Overall, I am very satisfied with my job as a CTC provider (JS6)	The term ‘CTC provider’ was replaced in each country with the context-appropriate term (i.e. HSAs, CHWs).
	(Mbindyo et al., 2009, Mutale et al., 2013, Bennett et al., 2001)	I am satisfied with the opportunities I have to use my abilities in my work (JS7)	
	Phase I findings and adapted from Mbindyo et al., 2009, Mutale et al., 2013, Prytherch et al., 2012, Bennett et al., 2001	I am satisfied that I accomplish something worthwhile with my work (JS8)	
	(Mbindyo et al., 2009, Mutale et al., 2013)	I think that my work as a CTC provider is not valuable these days (JS9)	Adapted from negatively worded item ‘I do not think that my work in this health facility is valuable these days’.
	Phase I findings	I am satisfied with the positive impact of my work (JS10)	
Work conscientiousness	(Mbindyo et al., 2009, Mutale et al., 2013, Bennett et al., 2001)	I can be relied upon at work (WC1)	Adapted from ‘I am reliable and dependable at work’.
	(Mbindyo et al., 2009, Mutale et al., 2013, Bennett et al., 2001)	I always complete my tasks efficiently and correctly (WC2)	
	(Mbindyo et al., 2009, Mutale et al., 2013, Bennett et al., 2001)	I take initiative to do things without being asked or told (WC3)	Adapted from ‘I do things that need doing without being asked or told’.
	(Mbindyo et al., 2009, Mutale et al., 2013, Bennett et al., 2001)	I am a hard worker (WC4)	
General motivation	(Bennett et al., 2001), adapted for/used in Mbindyo et al., 2009, Prytherch et al., 2012, Mutale et al., 2013, Penn-Kekana et al., 2005	These days I feel motivated to work as hard as I can (MOT1)	
	(Morrison et al., 2015)	I feel I am the right person to do this job (MOT2)	Adapted from ‘I am confident in my ability to do my job’.
Burnout	(Mbindyo et al., 2009, Mutale et al., 2013, Penn-Kekana et al., 2005)	When I get up in the morning I don’t feel like going to work (BO1)	Adapted from ‘Sometimes when I get up in the morning, I dread having to face another day at work’.
	(Mbindyo et al., 2009, Mutale et al., 2013, Penn-Kekana et al., 2005)	I feel emotionally tired at the end of the day (BO2)	
		I feel physically tired at the end of the day (BO3)	Added as connected to BO2.

*OC* organisational commitment, *JS* job satisfaction, *WC* work conscientiousness, *MOT* general motivation, *BO* burnout

### Phase III—Tool refinement and structure validation

The initial seven-factor model yielded negative residuals variance for two items of the burnout factor and a correlation factor > 1.00 between extrinsic and intrinsic satisfaction factors. Intrinsic and extrinsic items were therefore combined as a single, ten-item satisfaction factor, and the problematic burnout factor was removed. In the case of general motivation, the removal of a low-loading (< 0.3) item left only one item and the subsequent removal of this factor on the basis that a minimum of two items is required to constitute a factor [53]. Three additional items from the job satisfaction scale were also removed due to misspecification (negatively loading items and cross-factor loadings). Taken together, these modifications led to significant improvements in model fit (*χ*^2^ = 64, df = 48, *P* < 0.05, RMSEA = .032 (.000–.050); SRMR = .037; CFI = .975; TLI = .965) and to a revised 12-item, four-factor solution comprised of the following four factors: satisfaction (four items; M = 4.29, SD = 0.04), organisational commitment (two items; M = 4.17, SD = 0.15), community commitment (two items; M = 4.34, SD = 0.18), and work conscientiousness (four items; M = 4.28, SD = 0.04). Correlation coefficients between these factors demonstrated significant positive relationships, as summarised in Table 3. Composite reliability scores for each factor were .72, .53, .60, and .82 for satisfaction, organisational commitment, community commitment, and conscientiousness, respectively. The composite reliability score for the entire scale was CR = .90. Standardised and unstandardised factor loadings for the revised measurement model of the scale are reported in Table 4.

**Table 3:** Factor correlations for the initial version of the CTC Provider Motivational Indicator Scale

Factor	Organisational commitment	Job satisfaction	Community commitment	Conscientiousness
Organisational commitment	1.00			
Job satisfaction	0.73	1.00		
Community commitment	0.74	0.87	1.00	
Conscientiousness	0.57	0.77	0.75	1.00

**Table 4:** Standardised and unstandardised factor loadings (standard errors) for the initial version of the CTC Provider Motivational Indicator Scale, with a randomly selected sample of *n* = 345 CTC providers

Item	*β*	*B*	SE
Satisfaction			
I am satisfied that I accomplish something worthwhile with my work.	.53	1.00	–
I am satisfied with the positive impact of my work.	.62	1.09	.15
I am satisfied with the support I receive from my colleagues.	.63	1.03	.16
I am satisfied with the community thanks and recognition I receive for my work.	.72	1.40	.17
Organisational commitment			
I am proud to be working for my organisation as a CTC provider.	.78	1.00	–
My organisation really inspires me to do the very best on the job.	.39	.65	.14
Community commitment			
I am proud to be working for my community.	.73	1.00	–
My community inspires me to do the very best I can for them.	.58	1.04	.13
Work conscientiousness			
I can be relied upon at work.	.80	1.00	–
I always complete my tasks efficiently and correctly.	.71	.78	.08
I take initiatives to do things without being asked or told.	.69	.81	.07
I am a hard worker.	.73	.84	.08

All factor loadings are statistically significant (*P* < 0.05). *β* = standardised coefficient. *B* = unstandardised coefficient

This four-factor structure was subsequently validated using the second, randomly generated dataset, which was found to produce good fit statistics (*χ*^2^ = 78, df = 48, *P* < 0.05, RMSEA = .042 (.024–.059); SRMR = .039; CFI = .958; TLI = .942). Factor loadings for each observed variable on their respective latent variable were all statistically significant (*P* < .05) and positive. Composite reliability scores for each factor were .72, .56, .69, and .81 for satisfaction (M = 4.29, SD = 0.08), organisational commitment (M = 4.14, SD = 0.18), community commitment (M = 4.35, SD = 0.17), and conscientiousness (M = 4.26, SD = 0.05), respectively. Composite reliability scores for the combined 12 items was .90, indicative of good internal consistency. Correlation coefficients once again demonstrated positive relationships between factors, as summarised in Table 5. The final standardised and unstandardised factor loadings for the four-factor CTC Provider Motivational Indicator Scale are reported in Table 6.

**Table 5:** Factor correlations for the final version of the CTC Provider Motivational Indicator Scale

Factor	Organisational commitment	Job satisfaction	Community commitment	Conscientiousness
Organisational commitment	1.00			
Job satisfaction	0.68	1.00		
Community commitment	0.52	0.92	1.00	
Conscientiousness	0.53	0.76	0.66	1.00

**Table 6:** Standardised and unstandardised factor loadings (standard errors) for the final version of the CTC Provider Motivation Scale, with the remaining randomly selected sample of *n* = 350 CTC providers

Item	*β*	*B*	SE
Satisfaction			
I am satisfied that I accomplish something worthwhile with my work.	.63	1.00	–
I am satisfied with the positive impact of my work.	.68	1.21	.17
I am satisfied with the support I receive from my colleagues.	.51	0.93	.17
I am satisfied with the community thanks and recognition I receive for my work.	.68	1.19	.18
Organisational commitment			
I am proud to be working for my organisation as a CTC provider.	.91	1.00	–
My organisation really inspires me to do the very best on the job.	.27	.42	.16
Community commitment			
I am proud to be working for my community.	.85	1.00	–
My community inspires me to do the very best I can for them.	.59	.95	.11
Work conscientiousness			
I can be relied upon at work.	.71	1.00	–
I always complete my tasks efficiently and correctly.	.68	.88	.09
I take initiatives to do things without being asked or told.	.72	.95	.12
I am a hard worker.	.76	1.04	.09

All factor loadings are statistically significant (*P* < 0.05). *β* = standardised coefficient. *B* = unstandardised coefficient

## Discussion

In response to recent calls for more rigorous approaches to the development of scales for use in the study of health worker motivation [29, 36], the current study synthesises the results of focus group discussions and interviews conducted with relevant stakeholders from six LMICs, with extant CTC provider motivation measurement literature, and advanced latent variable modelling techniques to propose a 12-item CTC Provider Motivational Indicator Scale. Consistent with previous studies favouring a three-factor structure [37, 38] of health worker motivation, our findings support the inclusion of organisational commitment, job satisfaction, and work conscientiousness as observable indicators of motivation among CTC providers. Our findings further suggest the addition of a fourth, community commitment, sub-scale as an indicator of CTC provider motivation. The addition of this fourth factor may reflect key differences between CTC providers and formal health workers, whereby unlike formal health workers, CTC providers tend to be selected by the community and work in the communities where they come from [54].

Findings from phase I suggest that determinants or indicators of CTC provider motivation include strong organisational commitment, intrinsic and extrinsic job satisfaction, work conscientiousness, and community commitment. Organisational commitment, as an important contributor to CTC provider motivation, is consistent with the findings from previous studies suggesting creating a sense of connectedness and belongingness within health worker programmes, or feeling part of a team, as a key strategy to increase health worker motivation and satisfaction [2, 55]. Moreover, organisational commitment is recognised as being particularly important in the prevention of CTC provider turnover [21]. However, and while organisational commitment was also retained as a sub-scale in previous studies [19, 37], it should be noted that the item ‘My organisation really inspires me to do the very best on the job’ in the current study achieved low factor loadings across both samples in phase III. This could be due to the differences in what CTC providers understood as ‘the organisation’ (i.e. Ministry of Health, direct supervisors, or NGOs) across the various contexts, as identified during phase I. Future iterations of this scale may therefore want to consider being more specific in reference to ‘the organisation’ (i.e. health facilities) or consider testing the inclusion of alternative items as measures of organisational commitment.

The emergence of community commitment in phase II represents an important contribution to the development of existing measures of motivation and is consistent with other CTC provider literature, whereby community commitment and gaining community respect are both noted reasons why CTC providers volunteer for community health programmes in Burkina Faso [56], Ghana [57], Nepal [38], Uganda [2], and Kenya [58]. The importance of job satisfaction and community commitment in determining CTC provider motivation is also reported by Rahman et al. [59], with both factors presented in their framework for CTC provider retention in Bangladesh. As with previous scale development studies [19, 37], job satisfaction, as measured by CTC provider satisfaction with the opportunity to apply their skills and to accomplish something worthwhile in their work, emerged as an important indicator of motivation among CTC providers across the six contexts. The identification of work conscientiousness as a key indicator of motivation was also identified as one of the most commonly cited characteristics used to define motivated nurses and auxiliary nurse midwives in Nepal [38]. Consistent with other findings, our results further support the removal of burnout [19, 37, 39] and general motivation sub-scales [37]. This latter may be accounted for by the sub-scale containing only two items, which increases the likelihood of encountering identification problems within confirmatory factor analysis [53].

Theoretically, the four factors of the CTC Provider Motivation Indicator Scale are expected to be more strongly endorsed (i.e. be given a higher score) in the case of more motivated CTC providers and be given a lower score among less motivated or demotivated CTC providers. Practically, and given the multi-dimensional component of the scale, it is recommended that scores on each of the sub-scales are calculated separately for each of the four dimensions, either as a total sub-scale score or as a mean sub-scale score for use in the subsequent analysis [29]. Doing so will facilitate monitoring and allow for timely intervention, in the case where decreases in motivational indicator scores are detected. Alternatively, and as the results of the four-factor latent structure support the idea that the sum value approximates the true value of the latent variable, a motivation indicator score can also be quickly and easily obtained by calculating a sum total score. Whether direct or indirect measures of motivation, or some combination of both, are best suited for the management of CTC provider programmes should be explored in future studies.

The current study is not without limitations. Firstly, while the current study supports the factorial validity of the Motivational Indicator Scale, further validation efforts are required to ascertain its predictive (i.e. criterion), concurrent, and temporal stability. Specifically, longitudinal research should assess the ability of the Motivational Indicator Scale to predict performance-related outcomes, such as community health outcomes, quality of care, and CTC retention. Second, both commitment scores were found to have low composite reliability. While this is indicative of low reliability of these two sub-scales, calculating reliability coefficients for sub-scales with only two items is noted as problematic [60]. Third, it is possible that the observed findings from the factor analyses reflect the idiosyncratic responses from this specific heterogenous sample of CTC providers. Future research should investigate whether the scale is as valid and useful among more homogenous samples of CTC providers (i.e. CTC providers of the same sex, age group, and context). Fourth, generalisability analyses to test for measurement invariance across different sample subgroups other than CTC providers (i.e. nurses, doctors, mid-level cadres) are recommended to mitigate the risk that any future differences found across subgroups are due to actual differences in motivational indicators, rather than differences in the performance of the scale across subgroups [61]. Fifth, while careful steps were taken in the translation of the scales to ensure that no single item meaning was ‘lost in translation’, we acknowledge that this is always a risk with translation. Finally, responses provided in phases I and III may have been affected by social desirability bias, as indicated by the relatively high mean scores, particularly for questions pertaining to work conscientiousness.

## Conclusion

While the development of a highly motivated CTC provider workforce on its own is insufficient to address the shortage and inequitable distribution of health workers in LMICs, motivation, as a key determinant of CTC provider performance and retention, is an important element for health managers and programme implementers to consider. When combined with adequate training, regular supervision, and community recognition, the CTC Provider Motivational Indicator Scale offers an easy-to-administer tool that programme managers can use to monitor desired increases and address unwanted decreases in CTC provider motivation.

## Supplementary information

**Additional file 1.** Interviews and focus group discussions conducted per country, by informant type.

## Data Availability

The datasets used and/or analysed during the current study are available from the corresponding author on reasonable request.

## Abbreviations

CFA: Confirmatory factor analysis
CFI: Comparative Fit Index
CTC: Close-to-community
FGD: Focus group discussion
HRM: Human resource management
LMIC: Low- and middle-income country
NGO: Non-governmental organisation
TLI: Tucker-Lewis Index
RMSEA: Root mean square error of approximation
SRMR: Standard root mean square residual
WHO: World Health Organization
