# Supplementary material for: Measuring motivation among close-to-community health workers: developing the CTC Provider Motivational Indicator Scale across six countries
Source: Hum Resour Health. 2020 Aug 1;18:54. doi: 10.1186/s12960-020-00495-7 (PMC7395979; doi:10.1186/s12960-020-00495-7)
Supplement: Supplementary file 1 — Additional file 1. Interviews and focus group discussions conducted per country, by informant type. [file 12960_2020_495_MOESM1_ESM.docx]

**Additional file 1. Interviews and focus group discussions conducted per country, by informant type**

|  | **Ethiopia** | **Kenya** | **Malawi** | **Mozambique** | **Bangladesh** | **Indonesia** |
| --- | --- | --- | --- | --- | --- | --- |
| **CTC providers** | | | | | | |
| **FGDs** | HEWs - 6 | CHWs - 6 | HSAs - 3 |  |  | Village midwives & village nurses – 3 |
| **SSIs** | HEWs - 12 |  | HSAs - 8 | APEs - 18 | Formal CTCPs - 8  Informal CTCPs -16 | Village midwives & village nurses - 44 |
| **CTC provider supervisors, managers, other key informants** | | | | | | |
| **SSIs** | *Kebele* administrators - 3  Health centre in charges - 3  Delivery case team leaders - 3  HEP coordinators - 3  Regional HEP coordinator - 1  Zonal HEP coordinator - 1 | CHEWs - 16  SCHMT members - 3  Facility in-charges - 4  National level policy makers - 4 | District level staff - 13  Health centre in charges - 2  NGO staff - 9 | Health facility supervisors - 3  District supervisors - 2 | Paramedics - 2  Clinic Managers - 2  Counsellors - 2  Nurse - 1  Programme officer -1 | Heads of PHC or *Puskesmas* - 4  Midwife coordinators - 2  Heads of district MCH section - 2 |
| **Community members (clients of CTC providers)** | | | | | | |
| **FGDs** | Women - 6  Men - 2 | Community members - 4 | Women - 7  Volunteers - 6 | Mothers - 8 | Married women -8  Married men - 4 | Men - 2 |
| **SSIs** | Mothers - 12  TBAs - 6 | Community members - 10 | Mothers - 1  TBAs - 6  Traditional leaders - 3  Volunteers - 2 | Community leaders - 6 |  | Mothers - 39  TBAs - 8  Heads of village & heads of PKK - 17 |

APE = *Agentes polivalentes elementares* (elementary multipurpose agents); CHEW = community health extension worker; CHW = community health worker; CTCP= close-to-community provider; FGD = focus group discussion; FWA= family welfare assistant; HEP = health extension programme; HEW = health extension worker; HSA = health surveillance assistant; MCH = Maternal and child health; PHC = Primary health care; PKK = *Pembinaan Kesejahteraan Keluarga* (refers to the ‘family welfare movement’ – an Indonesian women’s organization); SSI = semi-structured interview; NGO = non-governmental organization; *Puskesmas* = sub-district community health centre; SCHMT = sub-county health management team; TBA = traditional birth attendant.
